# Genetic Characterization of Methicillin-Resistant Staphylococcus aureus Isolated From Diabetic Foot Ulcers in a Tertiary Care Hospital in Mysuru, South India

**DOI:** 10.7759/cureus.70605

**Published:** 2024-10-01

**Authors:** Veerabhadra Swamy GS, Mahadevaiah Neelambike Sumana, Yogeesh D Maheshwarappa, Rashmi P Mahale, Chinchana Eshwarappa Shylaja, Krishna Karthik, Supreeta R Shettar, G K Megha

**Affiliations:** 1 Clinical Microbiology, Jagadguru Sri Shivarathreeshwara (JSS) Medical College and Hospital, JSS Academy of Higher Education, Mysuru, IND

**Keywords:** diabetic foot ulcers (dfus), genetic characterization, meca, mecc, methicillin-resistant staphylococcus aureus (mrsa), real-time polymerase chain reaction (rt-pcr)

## Abstract

Background

Diabetic foot ulcers (DFUs) are common complications in diabetes patients, often leading to sepsis and leg amputation. Methicillin-resistant Staphylococcus aureus (MRSA) infections in DFUs pose challenges due to methicillin resistance with *mecA* and *mecC* genes. This study aims to assess the prevalence of MRSA in clinical isolates from DFUs, analyze the antibiogram of MRSA isolates, and detect the presence of the *mecA* and *mecC* genes among MRSA isolates.

Methodology

The isolated *S. aureus* colonies were identified and antimicrobial susceptibility was performed using the Vitek-2 Compact system. Methicillin resistance was also confirmed through the disc diffusion method. Confirmed methicillin-resistant isolates were subjected to real-time polymerase chain reaction (RT-PCR) to detect *mecA* and *mecC* genes.

Results

A total of 474 purulent samples from DFUs yielded 541 distinct isolates, comprising 201 gram-positive and 340 gram-negative organisms. Among the gram-positive organisms, *Staphylococcus* species predominated, with 79 *S. aureus* isolates, 34 of which were methicillin-resistant. All MRSA isolates (100%) were sensitive to tetracycline, linezolid, teicoplanin, and vancomycin, and 94% were sensitive to cotrimoxazole but least susceptible to ciprofloxacin and levofloxacin. RT-PCR confirmed the presence of *mecA* genes in all 34 isolates and *mecC* genes in three isolates.

Conclusions

The presence of *mecA* in all 34 MRSA isolates underscores consistent methicillin resistance. The co-occurrence of *mecA* and *mecC* in three isolates hints at genetic diversity. Two MRSA isolates positive for *mecC* were isolated from rural patients involved in farming and animal husbandry, suggesting an occupational risk. The third patient was from a non-rural area, indicating potential alternative transmission pathways warranting further investigation.

## Introduction

Diabetic foot ulcers (DFUs) are characterized as open wounds or sores on the foot, resulting from complications related to neuropathy in individuals with diabetes. These foot ulcers are observed in both type 1 and type 2 diabetic patients, manifesting at a prevalence ranging from 15% to 20% [1]. The microbial diversity in DFUs is affected by several factors, including wound depth, tissue perfusion, altered host humoral immunity, and geographic location. In severe DFU cases, if the patient is not treated immediately, it may even lead to sepsis and amputation or disarticulation of the infected diabetic foot. In such cases, adequate antibiotic therapy is required with broad-spectrum antibiotics [2,3].

DFU can be monomicrobial and polymicrobial, infected by both gram-positive and gram-negative organisms. Methicillin-resistant *Staphylococcus aureus* (MRSA) infection is common in DFUs and often takes a prolonged time to heal. MRSA infections have been linked to extended hospital admissions, which not only increase the cost of care but also raise the risk of acquiring additional nosocomial infections, thus increasing the likelihood of heightened morbidity and mortality [4,5]. DFUs caused by MRSA tend to result in more adverse outcomes compared to DFUs caused by methicillin-sensitive *Staphylococcus aureus* (MSSA). The resistance observed in MRSA is a direct consequence of the expression of penicillin-binding protein 2a (PBP2a), which is encoded by the *mecA* gene. The *mecA* gene is located on the staphylococcalchromosome cassette mec (SCCmec), a substantial mobile genetic entity that exhibits variations in size and gene composition across MRSA strains [6].

In recent times, a novel *mecA* homolog determinant called the *mecC* gene (which encodes PBP-2c) has been sporadically detected and linked to resistance within MRSA strains. However, definitive molecular evidence establishing the role of *mecC* in conferring beta-lactam resistance in MRSA remains elusive [7,8]. The *mecC* MRSA is identified in MRSA strains of humans in very small numbers and a wide variety of non-human hosts across Europe and Asia, including India [9]. India holds the title of the diabetes capital, with 41 million Indians affected, accounting for one in every five diabetics globally [10,11]. Additionally, it leads to an increased prevalence of diabetic complications, including DFUs. It is estimated that each year, US$8,659 (₹7,19,996) is needed to treat each DFU patient, and this could overwhelm the already poorly resourced healthcare systems of low-income regions [11].

To improve the management and prognosis of DFUs, as well as reduce their economic burden, upgrading treatment plans, determining patient risks, improving infection control procedures, and allocating resources are essential. It is important to continuously monitor the range of organisms infecting these ulcers, as well as their antimicrobial resistance patterns. Hence, to help fill this knowledge gap, this study aims to determine the rate of MRSA among the clinical isolates of DFUs, analyze the antibiogram of MRSA isolates, and detect the *mecA* and *mecC* genes among the clinical isolates of MRSA at a tertiary care hospital in Mysuru, South India.

## Materials and methods

This laboratory-based, prospective study was conducted at a tertiary care hospital in Mysuru, Karnataka, over a year. Pus samples/aspirates collected from patients with DFUs and submitted for routine culture and sensitivity were included in the study if they yielded growth of MRSA. Isolates from all age and sex groups with suspected DFU were considered for inclusion. Only MRSA isolates from cultured pus samples were included, while isolates sensitive to methicillin were excluded from the study. Comprehensive details of patient history and clinical findings were obtained from the hospital information system, laboratory registers, and case records of the patients. A similar data set has been used to study the genetic characterization of vancomycin-resistant *Enterococcus* isolated from DFUs in a tertiary care hospital in Mysuru, South India.

*Staphylococcus aureus* identification

The received pus samples were cultured on blood agar, MacConkey agar, and colistin nalidixic acid media and incubated at appropriate conditions (37°C for 24 hours, aerobically). The suspected colonies were identified using microscopic examination, conventional biochemical tests, and an automated identification system (Vitek-2 Compact system, bioMérieux, France). The antimicrobial susceptibility test (AST) was also performed using the automated identification (Vitek-2 Compact system, bioMérieux, France) using P-628 panel drugs. All tests were performed and results were interpreted according to Clinical and Laboratory Standard Institute (CLSI) guidelines [12]. To assess the accuracy of the test results, internal quality control examinations were performed on culture media before the utilization of a new batch of materials. Internal quality control for pathogen identification and AST using the Vitek-2 Compact system was conducted using gram-positive ATCC strains (ATCC 25922 *S. aureus*, ATCC 29212 *Enterococcus faecalis*, and ATCC 25788 *Enterococcus casseliflavus*).

Screening of methicillin resistance among the identified *Staphylococcus aureus*

The isolated *S. aureus *samples were screened for methicillin resistance using the disc diffusion method on cation-adjusted Mueller-Hinton agar using a cefoxitin disc of strength 30 µg (SD041-HiMedia) and the automated system (Vitek-2 Compact system, bioMérieux, France). Both tests were performed and the results were interpreted according to CLSI guidelines [12].

Phenotypic detection of the *mecA* and *mecC* genes among *Staphylococcus aureus*

The organisms that were screened resistant for both cefoxitin and oxacillin in the Vitek-2 system were identified as *mecA* gene-producing MRSA. The organisms that were screened cefoxitin-resistant and oxacillin-sensitive in the Vitek-2 system were identified as *mecC* gene-producing MRSA [13].

Genotypic characterization of *Staphylococcus aureus*

All the *S. aureus* isolates were subjected to DNA extraction followed by *mecA* and *mecC* amplification using real-time polymerase chain reaction (RT-PCR) with *mecA* primers (F5’-TCCAGATTACAACTTCACCAGG-3’ forward primer amplifying 162 base pairs and F5’-GAAAAAAAGGCTTAGAACGCCTC-3’ reverse primer amplifying 138 base pairs) and *mecC* primers (R5’- CCACTTCATATCTTGTAACG-3’ forward primer amplifying 162 base pairs, and R5’- GAAGATCTTTTCCGTTTTCAGC-3’ reverse primer amplifying 138 base pairs). The primers were synthesized by Barcode Biosciences. All reactions were performed separately in a final volume of 25 µL in 96-well hard-shell PCR plates. Each primer pair was used separately for respective *mecA* and *mecC* gens. The reaction mixture (25 mL) consisted of 12.5 mL TB Green Premix Ex Taq II (Tli RNaseH Plus) (2×), 1 μL each of both forward and reverse primer (10 μM), 2 μL of template DNA (<100 ng), and 8.5 μL of sterile purified water. The amplification was performed with initial denaturation starting at 95°C for 30 seconds, followed by 39 (40) cycles of annealing at 95℃ for five seconds, and extension at 60°C for 30 seconds. The melting curve was performed at 65-95℃ for five seconds.

## Results

In this investigation, during the study period, the laboratory received a total of 474 clinical samples from patients with DFUs. Among the 474 samples, 402 (84.81%) exhibited microbial growth when subjected to culture. Within the subset of 402 samples that demonstrated positive growth, a majority of 249 (61.94%) samples resulted in monomicrobial growth. Conversely, 153 (38.08%) samples exhibited polymicrobial growth, contributing to 541 distinct isolates. Of the 541 distinct isolates, 201 (37.16%) were gram-positive organisms, while 340 (62.84%) were gram-negative organisms. Within the 201 gram-positive organisms, staphylococcal species dominated, with 115 (21.25%) isolates, followed by *Enterococcus* (N = 43, 7.94%) and streptococci (N = 43, 7.94%). Of a total of 115 staphylococcal species, 79 (68.6%) isolates were identified as *S. aureus*, and 34 of these *S. aureus* isolates were determined to be methicillin-resistant.

All 34 MRSA isolates were from patients diagnosed with type 2 diabetes mellitus. The highest incidence of MRSA was seen within the 51-60-year age group, accounting for 12 (35.29%) cases. The 41-50-year age group followed closely with 10 (29.41%) cases. Subsequently, the 61-70-year age group exhibited the next highest occurrence of nine (26.47%) cases. Notably, the median age of the subjects under investigation was 51-60 years. The male-to-female ratio in 34 MRSA isolates was 4:1, with 27 (79.41%) isolates from males and seven (20.58%) from female patients. Of the 34 MRSA strains isolated, the majority were from inpatients, accounting for 29 (85%) of the cases, and the remaining were isolated from patients attending outpatient clinics.

Antibiotic susceptibility pattern of methicillin-resistant *Staphylococcus aureus* isolates using the Vitek-2 system

All MRSA isolates exhibited sensitivity to linezolid, teicoplanin, tetracycline, vancomycin, and daptomycin, with a sensitivity rate of 100%. Additionally, 94.11% of isolates were sensitive to trimethoprim/sulfamethoxazole, while 70.58% showed sensitivity to gentamicin. The isolates demonstrated the least susceptibility to ciprofloxacin and levofloxacin, as indicated in Table 1.

**Table 1:** Antimicrobial susceptibility patterns of methicillin-resistant Staphylococcus aureus by the Vitek-2 method. Data are expressed as numbers (n) and percentages (%).

Drugs	Sensitive	Resistant
Cefoxitin	0 (0%)	34 (100%)
Oxacillin	5 (14.7%)	29 (85.3%)
Ciprofloxacin	6 (17.6%)	28 (82.4%)
Levofloxacin	6 (17.6%)	28 (82.4%)
Erythromycin	12 (35.2%)	22 (64.8%)
Clindamycin	17 (50%)	17 (50%)
Gentamycin	24 (70.5%)	10 (29.5%)
Trimethoprim/Sulfamethoxazole	32 (94.1%)	2 (5.9%)
Linezolid	34 (100%)	0 (0%)
Teicoplanin	34 (100%)	0 (0%)
Tetracycline	34 (100%)	0 (0%)
Vancomycin	34 (100%)	0 (0%)
Daptomycin	34 (100%)	0 (0%)

Comparative analysis of cefoxitin and oxacillin sensitivity

A total of 34 MRSA isolates were subjected to a disc diffusion test to determine their susceptibility to cefoxitin (30 µg). Both the Vitek-2 Compact system and disc diffusion method results were analyzed as per the CLSI guidelines [13]. All the isolates identified as MRSA by the Vitek-2 Compact system also showed resistance to cefoxitin by disc diffusion and no discordant results were found by both detection methods.

On comparison of oxacillin and cefoxitin susceptibility results, five (14.70%) MRSA isolates showed sensitivity to oxacillin using the Vitek 2 system but were resistant to cefoxitin on the disc diffusion test. All remaining isolates showed resistance to both oxacillin and cefoxitin.

Comparative analysis of molecular and genotypic identification methods

All MRSA isolates were subjected to DNA extraction as per the manufacturer’s instructions (Juniper Life Sciences Pvt. Ltd.) and followed RT-PCR through melt curve analysis to detect the *mecC* and *mecA* genes. The amplification process was performed on all 34 isolates and all 34 isolates that were identified as MRSA phenotypically were found to harbor the *mecA* (100%) gene, as revealed by RT-PCR. However, the *mecC* gene was detected in only three (8.82%) of 34 MRSA isolates.

Among the three *mecC* gene-positive isolates, two exhibited susceptibility to oxacillin while one isolate was resistant. Notably, strains carrying the *mecC* gene were isolated from individuals afflicted with DFUs and cellulitis, correlating community-associated MRSA (CA-MRSA). Interestingly, on contact tracing, two patients carrying the *mecC* gene were found to have occupations in agriculture and animal husbandry. They had constant contact with livestock (cows, buffalos, and sheep). Notably, another patient with the *mecC* gene in MRSA isolate was not from a rural area and did not have animal contact.

**Figure 1:**
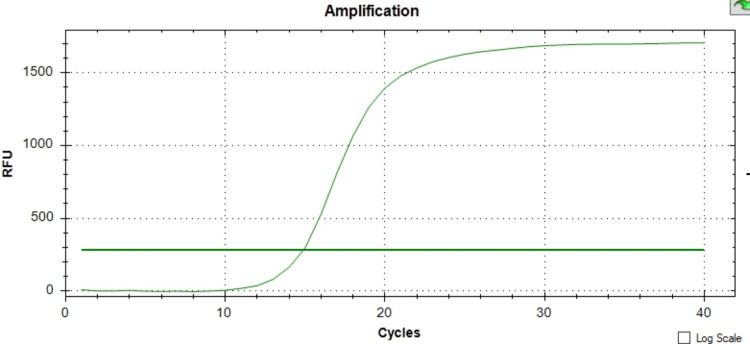
Amplification chart showing the sigmoid curve which indicates positivity for the presence of the mecA gene. RFU: relative fluorescence unit

**Figure 2:**
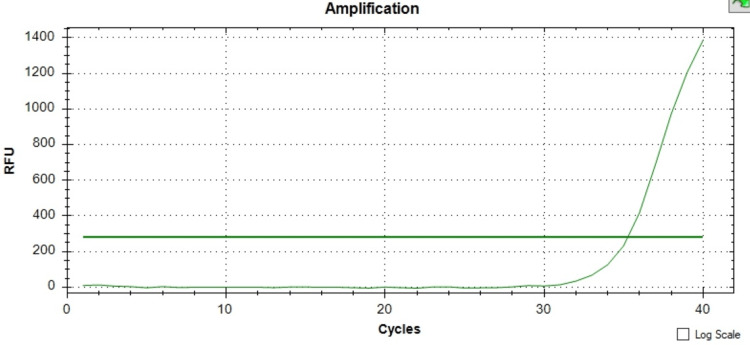
Amplification chart showing the sigmoid curve which indicates positivity for the presence of the mecC gene. RFU: relative fluorescence unit

**Figure 3:**
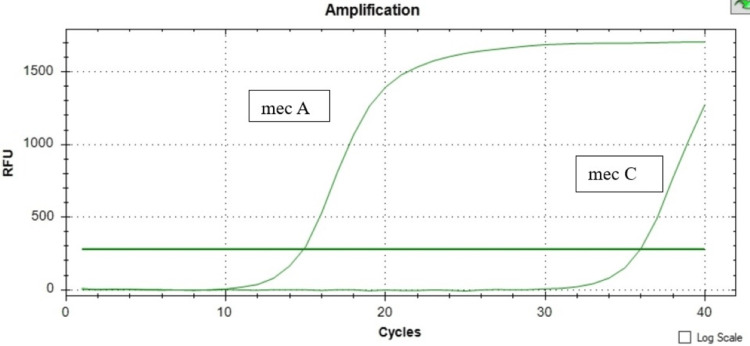
Amplification chart showing a number sigmoid curve which indicates the presence of the mecA and mecC gene. RFU: relative fluorescence unit

The presence of both the *mecA* and *mecC* genes was confirmed across all instances using the melt curve analysis revealing a single peak at the Tm of the product (for *mecA* positive control: Tm = 78°C, and for mecC positive control: Tm = 75°C) and with the peak height between 159 to 250 -d(RFU)/dT. For all *mecA* gene-positive isolates, the Tm range was between 77°C and 78°C, and for mec C gene-positive isolates, the Tm range was between 73°C and 74°C, as depicted in Figures 1-3.

## Discussion

The global rise in diabetes cases has contributed to a significant concern about the escalation of infections among diabetic patients [5]. This study sheds light on the multifaceted challenges posed by MRSA infections in DFUs within the context of increasing diabetic cases worldwide [14]. In this study, males 27 (79.41%) were affected predominantly by DFUs, while females accounted for seven (20.5%) of cases. These findings align with the studies conducted by Sekhar et al., Al Benwan et al., and Jain et al. [15-17]. The high prevalence of DFU among males can be attributed to increased engagement in outdoor activities and suboptimal adherence to foot care practices. Among the DFU cases, a significant majority of the patients were elderly, which is similar to the study conducted by Shekar et al. and Chavan et al. in Maharashtra [15,18]. In our investigation, we observed an extended duration of diabetes history among patients with DFU, averaging 13.08 ± 7.79 years. This finding closely parallels the study by Sekhar et al. [15], where the average duration was 18 years. In contrast, a study conducted by Chakraborty et al. [19] reported a significantly shorter average duration of five years.

Of the 474 clinical samples, 402 (84.8%) tested positive for bacterial growth, resulting in 541 distinct isolates, at a rate of 1.14 isolates per patient. This rate closely resembles the findings reported by Dawaiwala et al. (1.4 isolates per patient) [20]. However, studies by Gadepalli et al. (2.3) [21] and Selvarajan et al. (1.8) [22] have reported higher rates of bacterial isolation from DFUs. This could be because of suboptimal sample collection where colonizers might have contributed to the higher rate of bacterial isolation.

The predominant infectious agents observed in DFU samples seem to differ in the western part of the world. Notably, in our study, there was a higher prevalence of gram-negative organisms at 340 (62.84%) compared to gram-positive organisms at 201 (37.16%), which is consistent with major studies conducted in southern India [2,4,22] that emphasize the high prevalence of gram-negative pathogens. In contrast, gram-positive organisms predominate as causative agents in the western part of the world [14,23]. In our study, *S. aureus *(79, 14.60%) was the more predominant pathogen, of which 34 were methicillin-resistant, aligning with previous studies by Dawaiwala et al. and Wang et al. [5,20] In our study, MRSA accounted for approximately 6.2% of the total isolates. However, a separate investigation conducted by Hartemann-Heurtier et al. [24] exhibited a notably higher isolation rate (18%) of MRSA. Precise and prompt identification of methicillin resistance plays a crucial role in treating patients infected with *S. aureus*. The most frequently used phenotypic methods in the lab for identifying MRSA include disc diffusion using cefoxitin. However, it is worth noting that CLSI no longer recommends oxacillin disc diffusion for this purpose.

In this study, all 34 isolates that tested resistant to methicillin by the Vitek-2 system were identified as MRSA, and they also exhibited resistance to cefoxitin in the disc diffusion method. Among these 34 MRSA isolates, 29 (85.29%) showed resistance to oxacillin with a minimum inhibitory concentration (MIC) of ≥4 µg/mL, while five (14.50%) isolates exhibited sensitivity to oxacillin with an MIC of 0.5 µg/mL. This correlation between the outcomes of the cefoxitin disc diffusion test and the Vitek 2 system confirms the accurate identification of methicillin resistance. All MRSA isolates analyzed using the Vitek-2 system displayed sensitivity to linezolid, teicoplanin, tetracycline, vancomycin, and daptomycin (24, 100%). Additionally, there was high sensitivity to trimethoprim/sulfamethoxazole 32 (94.11%) and gentamycin 24 (70.58%), while the least susceptibility was observed for ciprofloxacin and levofloxacin (6, 17.64%). These findings are consistent with previously reported studies [25-27].

The current gold-standard method for identifying MRSA involves the detection of the *mecA* or *mecC* gene using PCR. The presence of the *mecA* gene was successfully detected in all 34 of these isolates by RT-PCR. Among these 34 isolates that tested positive for the *mecA* gene, 29 exhibited resistance to oxacillin, while the remaining five were considered susceptible based on assessments conducted using the Vitek-2 system. This finding is in line with a study conducted by Panda et al. [28] in a tertiary care hospital in Odisha, which documented that approximately 39% of the *S. aureus* isolates contained the *mecA* gene and were classified as MRSA.

The recent discovery of the *mecALGA251* (*mecC*) gene, housed within a novel SCCmec XI element within *S. aureus*, has added complexity to the detection of methicillin resistance. In our investigation, the *mecC* gene was identified in only 3 out of 34 isolates that tested positive for the *mecA* gene, constituting approximately 8.82% of the total examined isolates. The *mecC* gene is usually found in *S. aureus* strains harbored in pigs and livestock [17,24,27,28]. Contact tracing of *mecC*-positive patients revealed that they were from rural areas, and their occupation was farming and animal husbandry. They were in constant contact with livestock. Therefore, there is a high chance that these patients may have contracted *S. aureus* infection from livestock, suggesting an occupational risk. The third patient was from a non-rural area, indicating potential alternative transmission pathways warranting further investigation.

Limitations of the study

The study was conducted at a single tertiary care hospital in Mysuru, South India. This could limit the generalizability of the findings to a broader population, as the prevalence of microbial strains and antimicrobial resistance patterns may vary across different geographical regions and healthcare settings. The study included a relatively small sample size of 474 clinical samples over one year. A larger sample size and a longer study duration could provide more robust data and better represent the diversity of diabetic foot ulcer cases. While the study aimed to detect the *mecA* and *mecC* genes among MRSA isolates, it only focused on these specific genetic markers. Other factors contributing to antimicrobial resistance or virulence, such as other resistance genes (*erm(A)*, *erm(B)*, *erm(C)*, *tet(K)*, *tet(M)*, and *blaZ*) or mobile genetic elements, were not investigated, which could provide a more comprehensive understanding of MRSA strains circulating in DFUs. The study primarily focused on microbial identification and AST, with limited follow-up on patient outcomes and clinical data. Long-term follow-up and assessment of factors such as wound healing, amputation rates, and recurrence of infections would provide valuable insights into the clinical implications of MRSA infections in DFUs. The study was conducted in South India, where demographic characteristics, healthcare practices, and microbial ecology may differ from other regions or ethnic populations. Therefore, extrapolating the findings to other populations should be done cautiously, considering potential ethnic and regional variations.

## Conclusions

This study highlights the significant prevalence of MRSA in DFU infections, with 43% of *S. aureus* isolates confirmed as MRSA. Both phenotypic and genotypic methods demonstrated methicillin resistance, with all MRSA isolates carrying the *mecA* gene. The detection of *mecC*-positive MRSA suggests a risk of occupational transmission, underscoring the need for a One Health approach to control the spread of antimicrobial resistance between animals and humans. Based on the antibiotic susceptibility patterns, tetracycline, doxycycline, and cotrimoxazole can be considered for empiric therapy in hemodynamically stable DFU patients. Linezolid should be reserved for tuberculosis treatment, while vancomycin and teicoplanin should be used in cases of sepsis or severe infections. Addressing MRSA in DFUs requires careful antibiotic selection and broader strategies to mitigate antimicrobial resistance.
